# Endocrown: An Alternative Approach for Restoring Endodontically Treated Molars with Large Coronal Destruction

**DOI:** 10.1155/2018/1581952

**Published:** 2018-08-30

**Authors:** Houda Dogui, Feriel Abdelmalek, Adel Amor, Nabiha Douki

**Affiliations:** ^1^Department of Dental Medicine, Hospital Sahloul, Sousse, Faculty of Dental Medicine, Monastir, Tunisia; ^2^Laboratory of Research in Oral Health and Maxillo Facial Rehabilitation (LR12ES11), University of Monastir, Monastir, Tunisia; ^3^Faculty of Dental Medicine, University of Monastir, Monastir, Tunisia

## Abstract

Rehabilitation of endodontically treated molar still remains a challenge. After endodontic treatment, molars lost their mechanical characteristics. In fact, they became fragile and that is in relation with the removal of pulp and surrounding dentin tissues. Endocrown which is a single partial restoration could be considered as a good alternative for restoring molars having large coronal destruction and presenting endodontic treatment difficulties. Through this work, we discuss the indication and use of endocrown to replace single crowns with intraradicular retention and to present a clinical case report of an endocrown-type restoration, fabricated from lithium disilicate ceramic (IPS e.Max CAD) in a mandibular first molar with extensive coronal destruction.

## 1. Introduction

There is still an important challenge for most dentists before being optimistic about the rehabilitation of endodontically treated teeth with extensive coronal destruction. The biomechanical principles of retention and resistance are deteriorating [1]. The existing biomechanical changes due to root canal therapy and the degree of lost dental tissue lead clinicians to restorative treatment planning [2].

For the case of teeth heavily damaged by dental caries or fractures, a treatment with a total crown supported by a cast metal core has been suggested [3]. Yet, a root perforation and thinning of the root canal walls due to over preparation might happen after using intraradicular posts [4].

Moreover, the limitations to the use of intraradicular posts, such as calcified root canals, narrow canals, or a fracture of an instrument, have led dentists to think of other alternatives, as the use of endocrowns, an adhesive endodontic crown [5, 6].

This complete glass ceramic crown restoration was proposed in 1999 by Bindl and Mörmann as a substitute to the full post-and-core-supported crown; “endocrown” is a one-piece ceramic construction. This crown would be fixed to the internal walls of the pulp chamber and on the cavity margins to improve macromechanical retention and the use of adhesive cementation would also improve microretention [7].

The purpose of the present paper is to present a clinical case, in which an esthetic and conservative posterior endocrown was used to restore a mandibular molar that presented endodontic treatment and extensive coronal destruction. We will discuss through this work the indication and the use of endocrown.

## 2. Case Report

**Figure 1:**
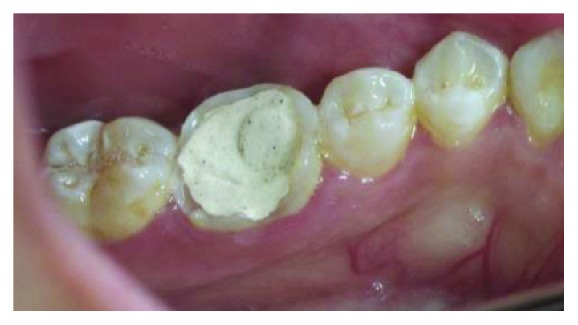
Clinical condition of tooth #46 with extensive glass ionomer cement restoration.

**Figure 2:**
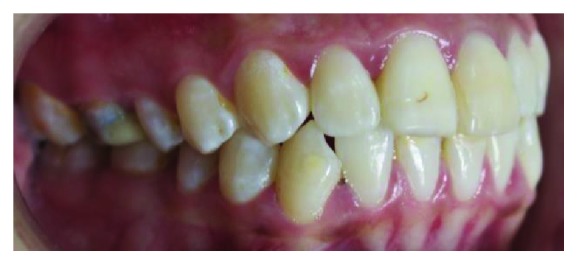
Relationship of crown height in occlusion with the antagonist tooth (side view).

**Figure 3:**
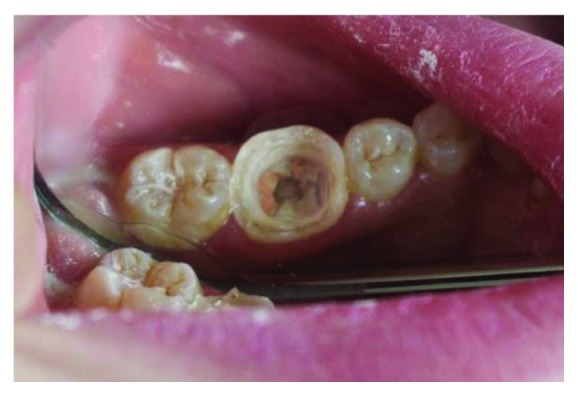
The molar after removal of the restoration.

A 23-year-old female was referred to our medicine dental department in UHC Sahloul, Sousse, for treatment of tooth #46. She suffered from major coronal destruction and needed to have her first molar restored. The medical history was noncontributory. Radiographic and clinical examinations were performed initially, and an extensive glass ionomer cement restoration of a nonvital tooth (46) was identified (Figures 1 and 2). The tooth was treated endodontically. The patient had an acceptable oral hygiene and a favorable occlusion. After removing the restoration, an endocrown restoration was recommended because of the amount of remaining tooth structure and the thickness of the walls (Figure 3). The prosthetic decision was to restore tooth (46) with an endocrown fabricated from lithium disilicate ceramic (IPS e.Max CAD). The preparation for the endocrown is different from the conventional complete crown. This monolithic, ceramic adhesive restoration requires specific preparation techniques to be suitable for especial biomechanical needs.

This is aimed at achieving achieve an overall reduction in the height of the occlusal surface of at least 2 mm in the axial direction and to get a cervical margin or “cervical sidewalk” in the form of a butt joint. The cervical margin has to be supragingival and enamel walls less than 2 mm have to be eliminated.

Differences in levels between the various parts of the cervical margin should be linked by a slope of no more than 60° to escape a staircase effect. We used a cylindrical-conical diamond bur held parallel to the occlusal plane, to reduce the occlusal surface. Then we used a diamond wheel bur to control the orientation of the reduction and to guarantee a flat surface thanks to its shape.

We used a cylindrical-conical diamond bur with a total occlusal convergence of 7° to create continuity between the coronal pulp chamber and endodontic access cavity. The bur was orientated along the long axis of the tooth; the preparation was done without too much pressure and without touching the pulpal floor.

Removing too much tissue from the pulp chamber walls will reduce their thickness and the width strip of enamel. The depth of the cavity must be at least 3 mm.

**Figure 4:**
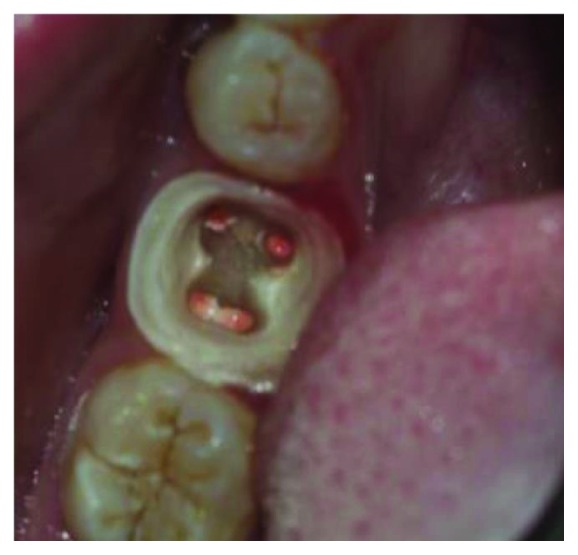
Removing Gutta-percha to a depth not exceeding 2 mm.

The entrance to the pulpal canal was opened. Gutta-percha was removed to a depth not exceeding 2 mm to profit from the saddle-like anatomy of the cavity floor. Nonabrasive instrument was required to maintain the integrity of the canal entrance. No drilling of dentin was carried out. The remaining tooth structure was still strong (Figure 4).

**Figure 5:**
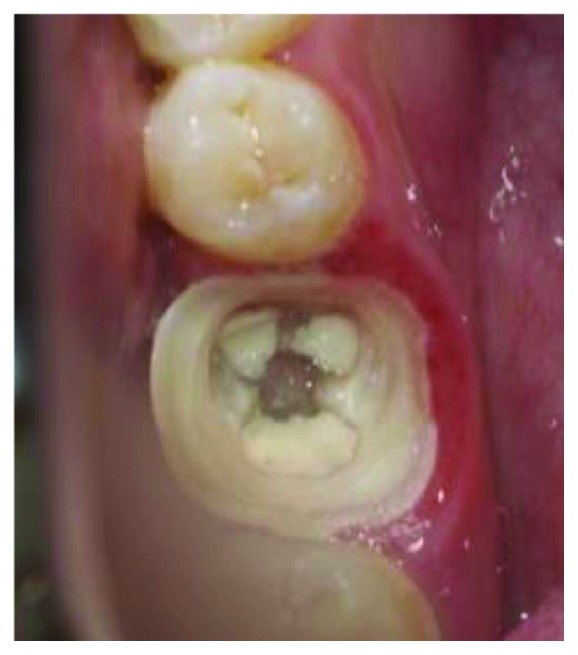
Lining the root canal entrances with glass ionomer cement.

We ended the preparation with lining the root canal entrances with glass ionomer cement to protect the orifice of the canal (Figure 5).

After evaluating the entire cavity and the interocclusal space, the impression of the tooth was taken by double impression technique using additional silicone. After visualization and analysis of the quality of the impression, we selected the ceramic shade and sent the impression to the laboratory.

**Figure 6:**
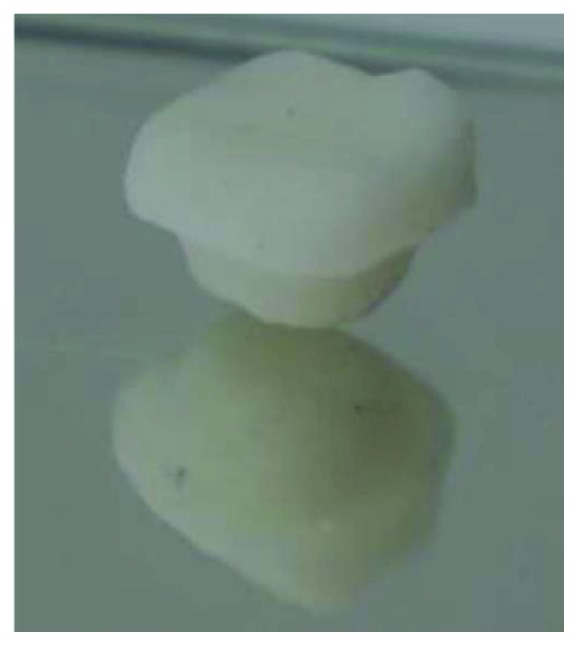
Aspect of the provisional restoration.

**Figure 7:**
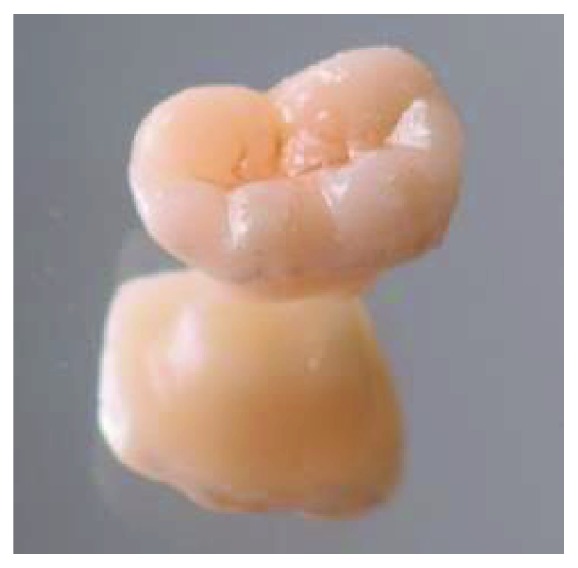
Aspect of the endocrown.

A provisional acrylic resin restoration was made by using block technic and cemented with eugenol-free temporary cement (Figure 6). The endocrown was fabricated in the laboratory using CAD-CAM technology and was positioned on the master cast (Figure 7).

Then we made a try-in of the endocrown and tested occlusion, internal, and proximal adjustments. Right after this, we sent it back to the laboratory for application of the colorant and glaze. In the following session, the internal surface of the endocrown was etched with hydrofluoric acid, rinsed with water, and dried with an air syringe. Next, a coat of a silane coupling agent was applied for 1 minute and dried.

Rubber dam was used to achieve proper isolation, and then phosphoric acid was applied onto the tooth surface for 15 sec on dentin and 30 sec on enamel, then abundantly washed and dried, applied with adhesive, and polymerized for 20 sec with light curing.

**Figure 8:**
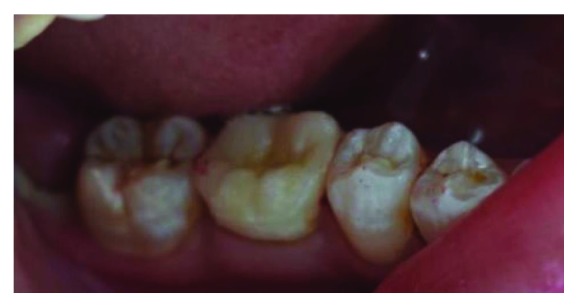
Try-in of the occlusion.

**Figure 9:**
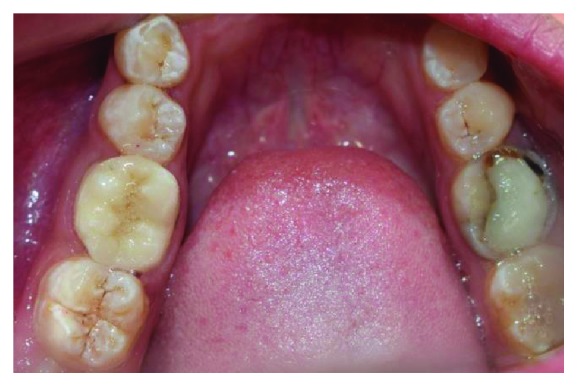
Final occlusal view after bonding the endocrown.

A thin layer of a dual polymerizing resin was applied to the prosthetic endocrown and then was inserted into the tooth and polymerized at intervals of 5 seconds, making it easy to remove cement excesses. After that, it was polymerized for 60 seconds on all surfaces. The restoration was examined for any occlusal interference using ceramic finishing instruments (Figure 8). The final restoration is shown in Figure 9.

## 3. Discussion

The project of the restorative treatment of molars with a large coronal destruction, a clinical challenge, requires careful planning. That is why the dentist has to decide for the best treatment option to ensure an efficient treatment providing clinical longevity of molars.

The endocrown is convenient for all molars, particularly those with clinically low crowns, calcified root canals, or narrow canals [8]. But it is not recommended if adhesion cannot be assured, if the pulpal chamber is less than 3 mm deep, or if the cervical margin is less than 2 mm wide for most of its circumference [9].

This has been shown to be an advantageous technique as the procedure is easy; it facilitates the steps of impression taking and protects the periodontium [8, 10]. Also, the use of ceramic has the advantages of biocompatibility and biomimicry and its wear coefficient is close to that of the natural tooth. Furthermore, the single interface of a 1-piece restoration makes cohesion look better [11, 12].

The objective of the preparation is to get a wide and stable surface resisting the compressive stresses that are frequent in molars [13]. The prepared surface is parallel to the occlusal plane to provide stress resistance along the major axis of the tooth [14]. The stress levels in teeth with endocrowns were lower than in teeth with prosthetic crowns [11, 15].

Due to the development of adhesive cementation systems, the need for macroretentive preparation for crowns has decreased [16].

The pulpal chamber cavity provides also retention and stability. Its trapezoidal shape in mandibular molars and triangular shape in maxillary molars increase the restoration's stability, and additional preparation is not needed. The saddle form of the pulpal floor increases stability. This anatomy, along with the adhesive qualities of the bonding material, makes it unessential to attempt further use of post-involving root canals [14]. In fact, the root canals do not need any specific shape; therefore, they are not fragilized by the drilling and they will not receive the stresses associated with the use of post [17]. The compressive stresses are reduced, being distributed over the cervical butt joint and the walls of the pulp chamber [8, 14, 18, 19].

In 2018, Dartora et al. have evaluated the biomechanical behavior of endodontically treated teeth restored using different extensions of endocrowns inside the pulp chamber; it has concluded that the greater extension of endocrowns provided better mechanical performance. A 5 mm extension presented lower intensity and a better stress distribution pattern than a 1 mm extension which presented a low fracture resistance and a high possibility of rotating the piece when in function [20, 21].

An in vitro study performed by Taha et al. was done to assess the effect of varying the margin designs on the fracture resistance of endodontically treated teeth restored with polymer-infiltrated ceramic endocrown restorations. The results showed that endocrowns with axial reduction and a shoulder finish line had higher mean fracture resistance values than endocrowns with butt margin design.

It has been also shown that butt joint designs provided a stable surface that resists the compressive stresses because it is prepared parallel to the occlusal plane [22].

In 2012, Biacchi and Basting compared the fracture strength of 2 types of full ceramic crowns: indirect conventional crowns retained by glass fibre posts and endocrowns. They came to the conclusion that endocrowns were more resistant to compressive forces than the first ones. More recently, finite element analysis highlighted the role of endocrowns in stress distribution [7].

According to Schultheis et al., endocrown seems to be a more reliable alternative for posterior loadbearing teeth, whereas a bilayer configuration is more susceptible to reduce load fracture failure [23].

As stated by Biacchi et al., endocrowns procure adequate function and esthetics and preserve the biomechanical integrity of nonvital posterior teeth. The restoration is reported to be less exposed to the adverse effects of degradation of the hybrid layer [24].

A research comparing equivalent stresses in molars restored with endocrowns as well as posts and cores during masticatory simulation using finite element analysis revealed that teeth restored by endocrowns are potentially more resistant to failure than those with FRC posts. This study also showed that under physiological loads, ceramic endocrowns ideally cemented in molars should not be damaged or debonded [15].

A systematic review achieved by Sedrez-Porto et al. has evaluated clinical (survival) and in vitro (fracture-strength) studies of endocrown restorations compared to conventional treatments using intraradicular posts, direct composite resin, or inlay/onlay restorations; it has been shown that endocrowns may perform similarly or better than the conventional treatments [25].

Altier et al. compared the fracture resistance of three different endocrowns made of lithium disilicate ceramic and two different indirect resin composites (Solidex composite and Gradia composite) and determined that lithium disilicate ceramic endocrowns exhibited higher fracture strength than the indirect composite groups [26].

It has been shown that endocrowns made of lithium disilicate-based ceramics are considered among the best restorative materials because of their adhesive properties; also, they promoted micromechanical interlocking with resin cement [7, 27].

An in vitro study accomplished by Gresnigt et al. evaluated the effect of axial and lateral forces on the strength of endocrowns made of Li2Si2O5 and multiphase resin composite. It has been concluded that under axial loading, both Li2Si2O5 and multiphase resin composite used as endocrown material presented similar fracture strength but under lateral forces, the latter exhibited significantly lower results [27].

In 2018, Tribst et al. evaluated the influence of a restorative material type on the biomechanical behavior of endocrown restorations and concluded that Leucite presents a better stress distribution and it can be a promising alternative to lithium disilicate for the manufacture of endocrown restorations [28].

Another research achieved by Skalskyi et al. compared the fracture resistance of different restorative materials used in dental endocrown restorations. It has demonstrated that the mechanical behavior of the restorative materials in the tooth restorations changed. The zirconium dioxide endocrowns cracked resulting to crack propagation in the tooth. It has been also shown that the use of metal ceramic as endocrown material may provide the lowest risk of failure during clinical use and had the highest fracture strength [29].

An investigation made by Darwish et al. showed that endodontically treated maxillary premolars restored with resin nanoceramic endocrowns presented better internal adaptation compared to those restored with lithium disilicate endocrowns and that endocrown preparation with smaller axial wall divergence (“6”degree) provided better internal fit [30].

In a recent study, Zoidis et al. proposed polyetheretherketone (PEEK) as an alternative framework material for endocrown restorations. They demonstrated that the elastic modulus of the polyetheretherketone framework (4 GPa) veneered with indirect composite resin could dampen the occlusal forces protecting tooth structures better than ceramic materials. But further long-term clinical evidence is required [31].

Recent scientific researches have focused on the positive results achieved during the last 12 years of endocrown restorations made with CEREC 3 and Vita Mark II feldspathic ceramic in a CAD-CAM system, with an estimated success of 90.5% for molars and 75% for premolars in 55 patients [1, 32].

According to Belleflamme et al., even in the presence of extensive coronal tissue loss or occlusal risk factors, such as bruxism or unfavorable occlusal relationships, endocrowns could be a reliable approach to restore severely damaged molars and premolars [33].

## 4. Conclusion

The preparation for endocrowns is simple and can be achieved quickly. Root canals are not engaged in the process, and the procedure is less traumatic than others. The supragingival position of the cervical margin protects the marginal periodontium, facilitates impression taking, and preserves the solid substance of the remaining tooth. Forces are dispersed over the cervical butt joint (compression) and axial walls (shear force), thus moderating the load on the pulpal floor.

The endocrown represents a very hopeful treatment alternative for endodontically treated molars, it allows maintaining of tooth structure, it is compatible with goal minimally invasive dentistry, and it is adequate for the concept of biointegration. It is a conservative approach for mechanical and aesthetic restoration of nonvital posterior teeth.

This type of reconstruction, which is still uncommon, should be more widely known and practised.

## References

[1] Ploumaki A., Bilkhair A., Tuna T., Stampf S., Strub J. R. (2013). Success rates of prosthetic restorations on endodontically treated teeth; a systematic review after 6 years. *Journal of Oral Rehabilitation*.

[2] Polesel A. (2014). Restoration of the endodontically treated posterior tooth. *Giornale Italiano di Endodonzia*.

[3] Stern N., Hirshfeld Z. (1973). Principles of preparing endodontically treated teeth for dowel and core restorations. *The Journal of Prosthetic Dentistry*.

[4] Asmussen E., Peutzfeldt A., Sahafi A. (2005). Finite element analysis of stresses in endodontically treated, dowel-restored teeth. *The Journal of Prosthetic Dentistry*.

[5] Bindl A., Mörmann W. H. (1999). Clinical evaluation of adhesively placed cerec endo-crowns after 2 years: preliminary results. *The Journal of Adhesive Dentistry*.

[6] Göhring T. N., Peters O. A. (2003). Restoration of endodontically treated teeth without posts. *American Journal of Dentistry*.

[7] Biacchi G. R., Basting R. T. (2012). Comparison of fracture strength of endocrowns and glass fiber post-retained conventional crowns. *Operative Dentistry*.

[8] Menezes-Silva R., Espinoza C. A. V., Atta M. T., Navarro M. F. L., Ishikiriama S. K., Mondelli R. F. L. (2016). Endocrown: a conservative approach. *Brazilian Dental Science*.

[9] Fages M., Bennaser B. (2013). The endocrown: a different type of all-ceramic reconstruction of molars. *Journal of the Canadian Dental Association*.

[10] Carlos R. B., Thomas Nainan M., Pradhan S., Sharma R., Benjamin S., Rose R. (2013). Restoration of endodontically treated molars using all ceramic endocrowns. *Case Reports in Dentistry*.

[11] Lin C. L., Chang Y. H., Chang C. Y., Pai C. A., Huang S. F. (2010). Finite element and Weibull analyses to estimate failure risks in the ceramic endocrown and classical crown for endodontically treated maxillary premolar. *European Journal of Oral Sciences*.

[12] Zarone F., Sorrentino R., Apicella D. (2006). Evaluation of the biomechanical behavior of maxillary central incisors restored by means of endocrowns compared to a natural tooth: a 3D static linear finite elements analysis. *Dental Materials*.

[13] Zogheib L. V., de Siqueira Ferreira Anzaloni Saavedra G., Cardoso P. E., Valera M. C., Araújo M. A. M. (2011). Resistance to compression of weakened roots subjected to different root reconstruction protocols. *Journal of Applied Oral Science*.

[14] Fages M., Bennasar B. (2013). The endocrown: a different type of all-ceramic reconstruction for molars. *Journal Canadian Dental Association*.

[15] Dejak B., Młotkowski A. (2013). 3D-finite element analysis of molars restored with endocrowns and posts during masticatory simulation. *Dental Materials*.

[16] Lander E., Dietschi D. (2008). Endocrowns: a clinical report. *Quintessence International*.

[17] Fernandes A. S., Dessai G. S. (2001). Factors affecting the fracture resistance of post-core reconstructed teeth: a review. *The International Journal of Prosthodontics*.

[18] Rocca G. T., Daher R., Saratti C. M. (2018). Restoration of severely damaged endodontically treated premolars: the influence of the endo-core length on marginal integrity and fatigue resistance of lithium disilicate CAD-CAM ceramic endocrowns. *Journal of Dentistry*.

[19] Fernandes da Cunha L., Mondelli J., Auersvald C. M. (2015). Endocrown with leucite-reinforced ceramic: case of restoration of endodontically treated teeth. *Case Reports in Dentistry*.

[20] Dartora N. R., de Conto Ferreira M. B., Moris I. C. M. (2018). Effect of intracoronal depth of teeth restored with endocrowns on fracture resistance: in vitro and 3-dimensional finite element analysis. *Journal of Endodontia*.

[21] Silva-Sousa Y., Gomes E. A., Dartora N. R. (2017). Mechanical behavior of endodontically treated teeth with different endocrowns extensions. *Dental Materials*.

[22] Taha D., Spintzyk S., Schille C. (2018). Fracture resistance and failure modes of polymer infiltrated ceramic endocrown restorations with variations in margin design and occlusal thickness. *Journal of Prosthodontic Research*.

[23] Schultheis S., Strub J. R., Gerds T. A., Guess P. C. (2013). Monolithic and bi-layer CAD/CAM lithium–disilicate versus metal–ceramic fixed dental prostheses: comparison of fracture loads and failure modes after fatigue. *Clinical Oral Investigations*.

[24] Biacchi G. R., Mello B., Basting R. T. (2013). The endocrown: an alternative approach for restoring extensively damaged molars. *Journal of Esthetic and Restorative Dentistry*.

[25] Sedrez-Porto J. A., de Oliveira da Rosa W. L., da Silva A. F., Münchow E. A., Pereira-Cenci T. (2016). Endocrown restorations: a systematic review and meta-analysis. *Journal of Dentistry*.

[26] Altier M., Erol F., Yildirim G., Dalkilic E. E. (2018). Fracture resistance and failure modes of lithium disilicate or composite endocrowns. *Nigerian Journal of Clinical Practice*.

[27] Gresnigt M. M. M., Özcan M., van den Houten M. L. A., Schipper L., Cune M. S. (2016). Fracture strength, failure type and Weibull characteristics of lithium disilicate and multiphase resin composite endocrowns under axial and lateral forces. *Dental Materials*.

[28] Tribst J. P. M., de Oliveira Dal Piva A. M., Madruga C. F. L. (2018). Endocrown restorations: influence of dental remnant and restorative material on stress distribution. *Dental Materials*.

[29] Skalskyi V., Makeev V., Stankevych O., Pavlychko R. (2018). Features of fracture of prosthetic toothendocrown constructions by means of acoustic emission analysis. *Dental Materials*.

[30] Darwish H. A., Morsi T. S., El Dimeery A. G. (2017). Internal fit of lithium disilicate and resin nanoceramic endocrowns with different preparation designs. *Future Dental Journal*.

[31] Zoidis P., Bakiri E., Polyzois G. (2017). Using modified polyetheretherketone (PEEK) as an alternative material for endocrown restorations: a short-term clinical report. *The Journal of Prosthetic Dentistry*.

[32] Otto T., Mörmann W. H. (2015). Clinical performance of chairside CAD/CAM feldspathic ceramic posterior shoulder crowns and endocrowns up to 12 years. *International Journal of Computerized Dentistry*.

[33] Belleflamme M. M., Geerts S. O., Louwette M. M., Grenade C. F., Vanheusden A. J., Mainjot A. K. (2017). No post-no core approach to restore severely damaged posterior teeth: an up to 10-year retrospective study of documented endocrown cases. *Journal of Dentistry*.

